# Functional Rescue of a Misfolded *Drosophila melanogaster* Dopamine Transporter Mutant Associated with a Sleepless Phenotype by Pharmacological Chaperones*^♦^

**DOI:** 10.1074/jbc.M116.737551

**Published:** 2016-08-01

**Authors:** Ameya Kasture, Ali El-Kasaby, Daniel Szöllősi, H. M. Mazhar Asjad, Alexandra Grimm, Thomas Stockner, Thomas Hummel, Michael Freissmuth, Sonja Sucic

**Affiliations:** From the ‡ Institute of Pharmacology, Center of Physiology and Pharmacology, Medical University of Vienna, A-1090 Vienna, Austria,; the § Department of Pharmacology, Faculty of Veterinary Medicine, Mansoura University, 35516 Mansoura, Egypt, and; the ¶ Department of Neurobiology, University of Vienna, A-1090 Vienna, Austria

**Keywords:** chaperone, dopamine, dopamine transporter, Drosophila, drug action, endoplasmic reticulum (ER)

## Abstract

Folding-defective mutants of the human dopamine transporter (DAT) cause a syndrome of infantile dystonia/parkinsonism. Here, we provide a proof-of-principle that the folding deficit is amenable to correction *in vivo* by two means, the cognate DAT ligand noribogaine and the HSP70 inhibitor, pifithrin-μ. We examined the *Drosophila melanogaster* (d) mutant dDAT-G108Q, which leads to a sleepless phenotype in flies harboring this mutation. Molecular dynamics simulations suggested an unstable structure of dDAT-G108Q consistent with a folding defect. This conjecture was verified; heterologously expressed dDAT-G108Q and the human (h) equivalent hDAT-G140Q were retained in the endoplasmic reticulum in a complex with endogenous folding sensors (calnexin and HSP70-1A). Incubation of the cells with noribogaine (a DAT ligand selective for the inward-facing state) and/or pifithrin-μ (an HSP70 inhibitor) restored folding of, and hence dopamine transport by, dDAT-G108Q and hDAT-G140Q. The mutated versions of DAT were confined to the cell bodies of the dopaminergic neurons in the fly brain and failed to reach the axonal compartments. Axonal delivery was restored, and sleep time was increased to normal length (from 300 to 1000 min/day) if the dDAT-G108Q-expressing flies were treated with noribogaine and/or pifithrin-μ. Rescuing misfolded versions of DAT by pharmacochaperoning is of therapeutic interest; it may provide opportunities to remedy disorders arising from folding-defective mutants of human DAT and of other related SLC6 transporters.

## Introduction

The dopamine transporter (DAT)^4^ is a member of the solute carrier 6 (SLC6) protein family. The main physiological role of DAT (SLC6A3) is to clear catecholamines (*i.e.* dopamine and norepinephrine) from the synaptic cleft to achieve rapid termination of neurotransmission. DAT operates in relay with the cytosolic vesicular monoamine transporter to replenish vesicular neurotransmitter stores and prepare them for subsequent release. DAT is a target of therapeutically relevant (*e.g.* methylphenidate) and illicit recreational drugs (*e.g.* cocaine, amphetamines, and cathinones) (1). Because the activity of DAT shapes dopaminergic neurotransmission, its activity has implications for several psychiatric disorders ranging from schizophrenia to attention-deficit hyperactivity disorder. Recently, several point mutations have been identified in patients suffering from infantile dystonia/Parkinson's disease (2–4). When mapped onto a structural model, these mutations are distributed in a seemingly random fashion over the protein, but closer inspection suggests that many are located at the lipid-protein interface (5). The vast majority of these mutations result in a folding defect, *i.e.* the translated protein is retained in the endoplasmic reticulum (ER) in an inactive state (2, 3).

Folding of proteins can be assisted by small molecules. These are thought to act in a manner analogous to proteinaceous chaperones; they bind to folding intermediates and lower the energy barrier for the transition into a trajectory that is conducive for formation of the fully folded state. The pharmacological chaperoning action of these cognate ligands is hence referred to as pharmacochaperoning (5) or pharmacoperoning (6). Alternatively, folding can be assisted by manipulating the proteinaceous chaperone machinery, which curtails the folding trajectory (7, 8). The folding trajectory of DAT is not understood, but several folding-deficient mutants of the closely related transporter serotonin transporter (SERT) have been examined: they can be rescued by pharmacochaperoning with ibogaine or noribogaine (8–10), which bind to the inward-facing transporter conformation (11, 12). In addition, several folding-deficient mutants can be rescued by heat-shock protein inhibitors (8).

A phenotype of reduced sleep was identified in fruit flies (*Drosophila melanogaster*), which had been subjected to random mutagenesis, and linked to a point mutation in the gene encoding *Drosophila* DAT (dDAT) (13). The mutation substitutes glycine 108 by glutamine and phenocopies the effect caused by deletion of DAT in flies, which are referred to as fumin (= sleepless) flies (14). This suggests that dDAT-G108Q is a loss-of-function mutation. Gly^108^ is located in the first intracellular loop (IL-1). In SERT, IL-1 interacts with the C terminus via a salt bridge to stabilize the folded structure (10). An analogous mechanism may operate in dDAT, because the crystal structure of dDAT visualizes a cation-π interaction between a Trp in the C terminus and an Arg in IL-1 (15). Accordingly, it appears justified to surmise that dDAT-G108Q has a folding defect. Here, we show that this is the case. In addition, we document that this folding defect of dDAT-G108Q can be remedied by pharmacochaperoning. The magnitude of the effect sufficed to restore sleep in flies expressing dDAT-G108Q.

## Results

### 

#### 

##### Effects of the G108Q Mutation on the Folding of dDAT

**FIGURE 1.**
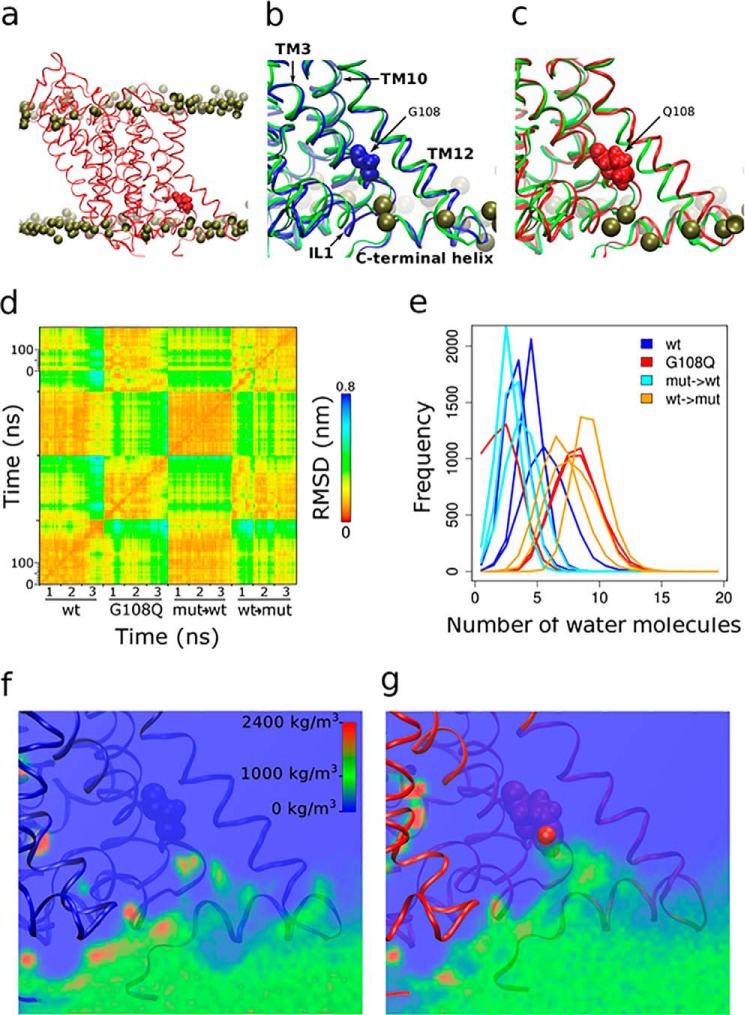
**Structural effect of the G108Q mutation probed by molecular dynamics simulations of dDAT.** *a, ribbon* diagram of membrane-inserted dDAT highlighting Gln^108^ in a space-filling representation. The phosphate atoms of the membrane lipid head groups are shown as *dark spheres. b* and *c,* representative computed structures of wild-type dDAT (*b*) and dDAT-G108Q (*c*). The computed backbone traces (*blue* in *b* and *red* in *c*) are compared with that of dDAT in the crystal structure (*green*) to illustrate the displacement of TM12 in the dDAT-G108Q mutant. *d,* r.m.s.d. matrix of 12 simulations represented as a heat map: three each of wild-type dDAT (WT) and of dDAT-G108Q (G108Q), three each of dDAT-G108Q mutated to wild-type DAT (mut → WT) and of wild-type dDAT mutated to dDAT-G108Q (WT → mut) after membrane insertion. The r.m.s.d. was calculated for the Cα atoms of the distal segment of TM12 starting with Gly^565^ to Thr^582^ for every frame (20 ps) of the 100-ns simulation. The *x* and *y* axes are identical; hence the plot allows for comparison of each TM12 structure (*i.e.* its r.m.s.d. deviation) with itself and with all other structures. *e,* number of water molecules within a 1.0-nm distance from the Cα atom of residue 108 was calculated in six independent simulations each of dDAT-G108Q (*orange* and *red traces*) and of wild-type DAT (*light* and *dark blue traces*). In five out of six simulations of dDAT-G108Q, the number of water molecules was increased when compared with wild-type dDAT. *f* and *g,* average water density is displayed as a heat map on a plane, which transects the nitrogen atom (*large red sphere* in *g*) of Gln^108^. This plane was placed at the identical position in wild-type dDAT.

Gly^108^ is located in the transmembrane region of dDAT in the interface between transmembrane (TM) helices TM3 and TM12. The dDAT-G108Q mutation introduces a large side chain into the helix interaction motif G*XXX*G of TM3 at the interface to TM12 (*cf.* Fig. 1*a*). We employed molecular dynamics (MD) simulations to examine the structural effect of the large hydrophilic side chain by using the crystal structure of dDAT (Protein Data Bank code 4XP1; compare Refs. 15, 16) as a template. The model of dDAT was built using MODELLER (17) by first reverting the thermo-stabilizing substitutions (V74A and L415A) of the crystallized species to their wild-type counterpart. The conformation of the second extracellular loop (EL2) of the crystal structure was replaced with that developed for the human DAT (18) because the EL2 of the crystal was unstable if its interactions with the co-crystallized antibody in the adjacent crystallographic unit were removed. The best three models of wild-type dDAT and dDAT-G108Q (out of 100 each) were inserted into a pre-equilibrated POPC membrane. Each system was independently equilibrated and then simulated for 100 ns. As a control for a possible bias, which could have been introduced by model selection or system assembly, we also changed the amino acid of residue 108 after membrane insertion. This resulted in a total of 12 simulations (*cf*. Fig. 1*d*). The pertinent observations can be summarized as follows: glutamine was not tolerated at position 108; its presence consistently caused a displacement of the second part of TM12 (*cf.* Fig. 1, *b* and *c*). Helix TM12 has a conserved proline in the center of the membrane bilayer resulting in a prominent kink, which divides TM12 into two segments. The C-terminal part of TM12 was pushed away from TM3 in all six simulations carrying the dDAT-G108Q mutation. We quantified the movement of the C-terminal part of TM12 by calculating a root mean square displacement (r.m.s.d.) matrix over all 12 MD simulations. The three wild-type simulations and the three reverted wild-type simulations showed small r.m.s.d. values throughout the trajectory and also relative to each other, indicating a stable conformation of TM12 (WT and mut → WT in Fig. 1*d*). In contrast, the MD simulations of the dDAT-G108Q mutant and the reverted dDAT-G108Q mutant showed higher r.m.s.d. values with respect to the starting structure, with respect to each other and in particular with respect to the six MD simulations of wild-type dDAT (G108Q and WT → mut in Fig. 1*d*). These movements indicate that TM12 fails to adopt a stable conformation in dDAT-G108Q.

The side chain of glutamine is polar and preferentially hydrated. We observed a local deformation of the membrane next to residue dDAT-G108Q. In addition, five out of six MD simulations showed water permeation through the deformed membrane and hydration of the side chain of Gln^108^. We quantified this observation by counting the average number of water molecules within 1.0 nm of the Cα atom of residue 108. The average number of waters was twice as high in the dDAT-G108Q mutants as in wild-type DAT (Fig. 1*e*). The water density was visualized in a slice, which was placed through the peak of the permeating water in DAT-G108Q (Fig. 1*g*) and the equivalent position of the wild-type dDAT (Fig. 1*f*). This slice illustrates the higher water density in the vicinity of residue 108 in dDAT-G108Q (*cf*. Fig. 1, *f* and *g*).

##### Misfolding and ER Retention of dDAT-G108Q and of hDAT-G140Q

**FIGURE 2.**
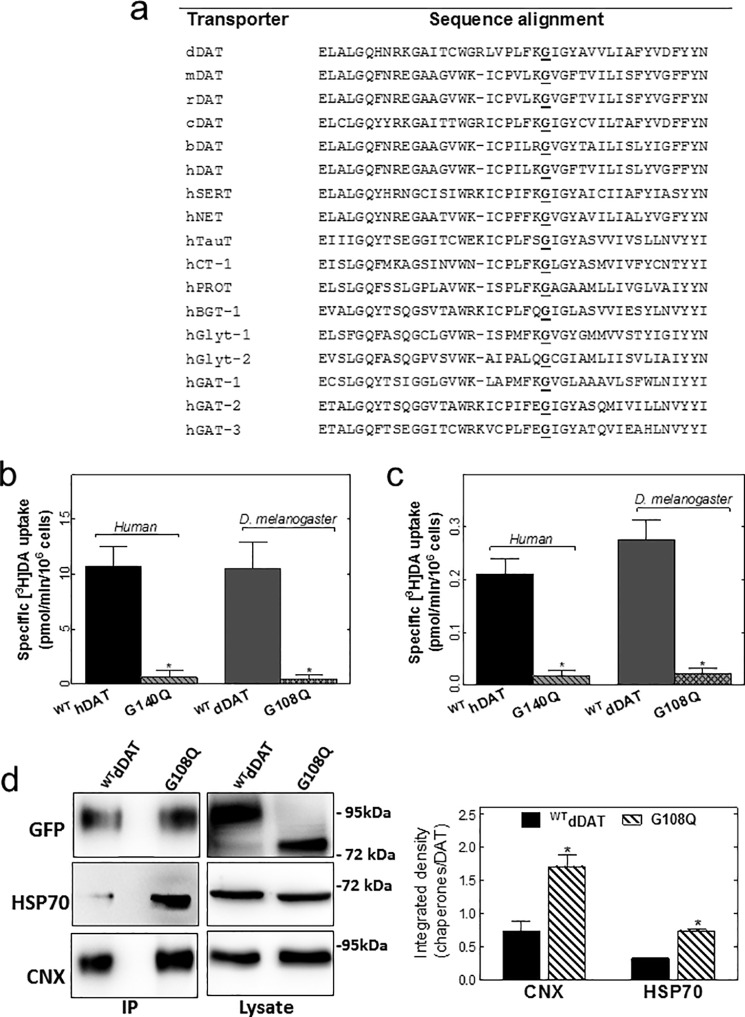
**Point mutation G108Q in dDAT and its human equivalent hDAT-G140Q gives rise to an ER-retained non-functional protein.** *a,* sequence alignment of the first intracellular loop and of segments of the adjacent transmembrane domains 1 and 2 starting with residue Glu^84^ in dDAT. The glycine residue at the 108th position in the dDAT is conserved in species orthologs (*hDAT,* human DAT; *mDAT,* murine DAT; *cDAT,* canine DAT; *bDAT,* bovine bDAT) and in other members of the SLC6 family (illustrated for the human orthologs). *b* and *c,* specific uptake of [^3^H]dopamine was determined as described under “Experimental Procedures” in HEK293 cells (*b*) and S2 cells (*c*) transiently transfected with plasmids driving the expression of YFP-tagged wild-type (*wt*) hDAT and dDAT as a reference and dDAT-G108Q and hDAT-G140Q. Data are means ± S.E. (*error bars*) from three independent experiments carried out in triplicate. The statistical comparison was done by *t* test (*, *p* < 0.05 significantly different from wild-type control). *d,* YFP-tagged wild-type (*wt*) dDAT and dDAT-G108Q were immunoprecipitated (*IP*) using an antibody directed against GFP from detergent lysates prepared from stably transfected HEK293 cells (see “Experimental Procedures”). Aliquots from the lysates (*right-hand blots*) and from the immunoprecipitates (*left-hand blots*) were resolved by SDS-PAGE. After transfer of the proteins to nitrocellulose, immunoreactive bands were visualized with anti-GFP, anti-calnexin, and anti-HSP70-1A antibodies and densitometrically quantified using the ImageJ software. The immunoblots shown are representative of three independent experiments, the quantification of which is shown in the *bar diagram*, where the amount of co-immunoprecipitated chaperones was related to the immunoprecipitated DAT (*error bars* represent S.E.). The statistical comparison was done by *t* test (*, *p* < 0.05, significantly different from wild type).

**FIGURE 3.**
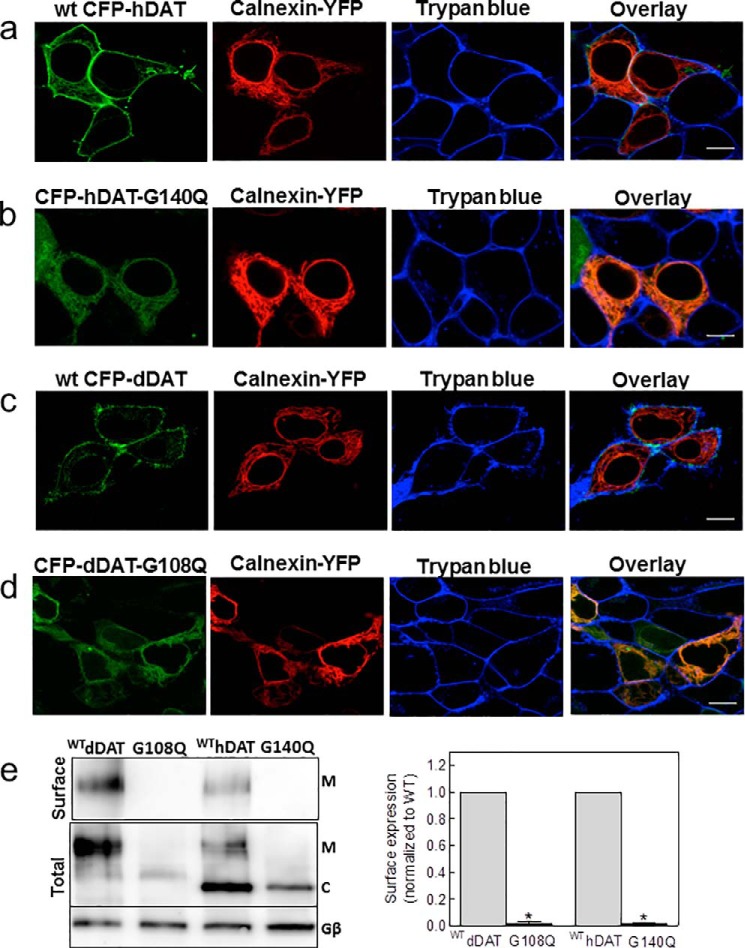
**Mutation of the conserved glycine residue results in ER retention of DAT.** HEK293 cells were transfected with plasmids encoding wild-type CFP-hDAT (*a*), CFP-hDAT-G140Q (*b*), wild-type CFP-dDAT (*c*), and CFP-dDAT-G108Q (*d*), all shown in *green.* After 24 h, the transfected cells were seeded onto poly-d-lysine-coated ibidi® glass bottom chambers. Confocal microscopy images were captured after an additional 24 h. Overlay images were produced to show co-localization between the different signals. A plasmid encoding YFP-calnexin was used to label the ER compartment (shown in *red*), and trypan blue (0.04% in PBS, shown in *blue*) was used to visualize the plasma membrane in the cells. *Scale bars* represent 10 μm. *e,* cell surface biotinylation was performed in HEK cells expressing the indicated DAT versions. *Top panel*, surface expression assessed by affinity precipitation of the transporter using streptavidin beads followed by immunoblotting; *bottom panels*, total lysate fractions and G protein β-subunit (as a loading control). The integrated intensity of the biotinylated bands (*M*) was quantified by ImageJ relative to the total amount of DAT immunoreactivity in the lysate (*i.e.* the bands with mature (*M*) glycosylation and core (*C*) glycosylation and normalized to wild type. The data are means ± S.E. from four independent experiments (*, *p* < 0.05, significantly different from corresponding wild type as assessed by a *t* test for unpaired data using the original rather than the normalized values).

Gly^108^ of dDAT is conserved among all species examined and is also found in all members of the SLC6 family (except for SLC6A10, *i.e.* creatine transporter 2, which is considered a pseudogene) (Fig. 2*a*). This strict conservation suggests an important role, possibly in protein folding. This conjecture was also supported by the molecular dynamics simulations summarized in Fig. 1. We verified this hypothesis by transiently expressing dDAT-G108Q and its human equivalent hDAT-G140Q in human embryonic kidney 293 cells (HEK293; Fig. 2*b*) and the *Drosophila* Schneider 2 cell line (*S2 cells*; Fig. 2*c*). Cell numbers were comparable after transient transfection of plasmids encoding wild-type and mutant transporters indicating that the transient expression of the mutated transporters did not affect cell viability. In contrast to the expression of the wild-type versions of DAT, the expression of the mutant proteins did not result in any measurable [^3^H]dopamine uptake (Fig. 2, *b* and *c*). Lysates prepared from stably transfected HEK293 cells only contained the ER resident core-glycosylated dDAT-G108Q, whereas the bulk of wild-type DAT was in the mature glycosylated form (*upper left-hand immunoblot* in Fig. 2*d*). Folding-deficient mutants of SERT are trapped in the ER in complex with the luminal chaperone calnexin and/or the cytosolic chaperone HSP70-1A (8, 10). Accordingly, we immunoprecipitated dDAT-G108Q to explore its association with calnexin and HSP70-1A; immunoprecipitates of dDAT-G108Q contained substantially higher levels of HSP70-1A and of calnexin than wild-type dDAT (*left-hand immunoblots* and *bar diagram* in Fig. 2*d*), whereas the detergent lysates contained comparable amounts of these chaperones (*upper right-hand immunoblot* in Fig. 2*d*). We also visualized the retention of both hDAT-G140Q (Fig. 3*b*) and dDAT-G108Q (Fig. 3*d*) in the endoplasmic reticulum by confocal microscopy; the CFP-tagged mutant transporters were co-localized with YFP-tagged calnexin, but they did not reach the cell membrane, which was delineated by staining with trypan blue. In contrast, the corresponding wild-type hDAT (Fig. 3*a*) and dDAT (Fig. 3*c*) reached the cell surface. These observations were confirmed by cell surface biotinylation experiments (Fig. 3*e*).

##### Rescue of DAT Mutants by Pharmacochaperoning

**FIGURE 4.**
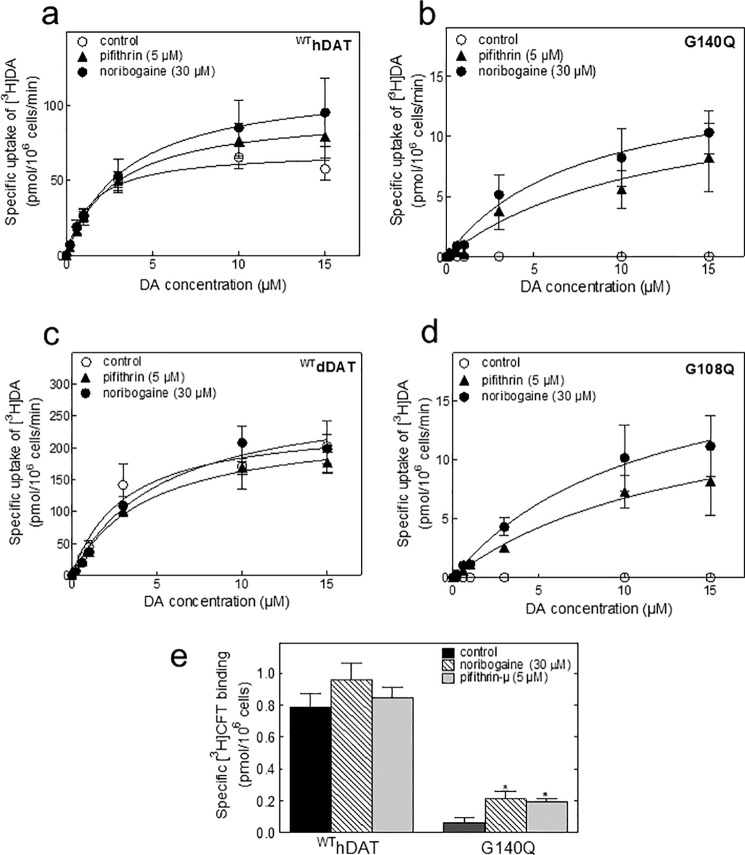
**Non-functional mutants dDAT-G140Q and hDAT-G108Q display specific dopamine uptake upon pharmacochaperoning.** HEK293 cells expressing wild-type hDAT (*a*), hDAT-G140Q (*b*), wild-type dDAT (*c*), and dDAT-G108Q (*d*) were seeded onto 48-well plates 24 h following the transfections. The cells were treated with noribogaine (30 μm) or pifithrin-μ (5 μm) for 24 h, prior to performing uptake assays. Specific [^3^H]dopamine uptake was carried out using the indicated concentrations of dopamine. Mazindol (30 μm) was used to determine nonspecific uptake levels, as outlined under “Experimental Procedures.” The data were obtained from three independent experiments, done in triplicate; the *error bars* indicate S.E. *e,* binding of [^3^H]CFT to intact HEK293 cells transiently expressing wild-type hDAT and hDAT-G140Q, treated with noribogaine (30 μm) or pifithrin-μ (5 μm) for 24 h prior to the binding assays. Shown are data that were obtained in four independent experiments carried out in parallel for wild-type and mutant DAT. The data are means ± S.E. (*n* = 4); the statistical comparison was done by paired *t* test with Bonferroni correction (*, *p* < 0.01, significantly different from untreated control values).

The plant-derived alkaloid ibogaine binds to the inward-facing conformation of SERT and DAT (11, 12). Noribogaine is a more potent analog. Both ibogaine (9) and noribogaine (8, 10) rescue misfolded SERT mutants. Accordingly, we tested whether noribogaine also rescued dDAT-G108Q and hDAT-G140Q; stably transfected HEK293 cells were preincubated with noribogaine for 24 h and removed by extensive washing, and dopamine uptake was measured subsequently (Fig. 4). In the absence of this pretreatment, there was no measurable specific uptake of [^3^H]dopamine (*open circles* in Fig. 4, *b* and *d, cf.* Fig. 2*b*). In contrast, uptake of [^3^H]dopamine was detectable in cells that had been incubated with noribogaine (*closed circles* in Fig. 4, *b* and *d*). Pifithrin-μ is a cell-permeable small molecule inhibitor of HSP70 (19). The action of noribogaine was recapitulated by pifithrin-μ (*triangles* in Fig. 4, *b* and *d*). Preincubation with either noribogaine or pifithrin-μ also, albeit only moderately, augmented uptake by cells expressing the pertinent wild-type transporters, *i.e.* hDAT (Fig. 4*a*) and dDAT (Fig. 4*c*). In addition, we verified these findings by measuring 2-β-carbomethoxy-3-β-(4-fluorophenyl)-*N*-[^3^H]methyltropane binding in intact cells expressing wild-type hDAT or the G140Q mutant (Fig. 4*e*). *Drosophila* DATs were not examined, because the affinity of cocaine and its analogs for dDAT is substantially lower than for the human ortholog (20). It is evident from the paired comparisons that only a fraction of wild-type transport (*cf. a* and *b* and *c* and *d* in Fig. 4) and binding (Fig. 4*e*) was restored by pharmacochaperoning.

**FIGURE 5.**
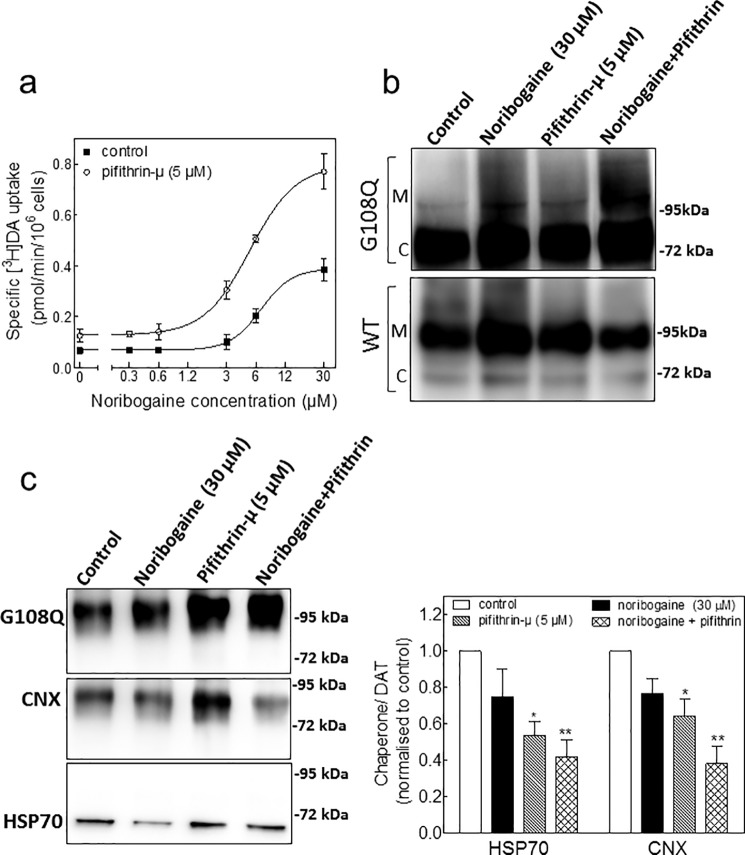
**Additive effect of pifithrin-μ and noribogaine in rescuing folding-deficient dDAT-G108Q and reducing the association of dDAT-G108Q with HSP70 and calnexin.** *a,* HEK293 cells stably expressing CFP-tagged dDAT-G108Q were seeded onto 48-well plates and incubated in the absence (control, *closed squares*) and presence of pifithrin-μ (5 μm; *open circles*) with increasing concentrations of noribogaine for 24 h. Transporter function was quantified by measuring specific [^3^H]dopamine uptake. Data are from three independent experiments done in triplicate; *error bars* indicate S.E. The curves were generated by fitting the data points to the equation of a rectangular hyperbola. *b,* detergent lysates were prepared from HEK293 cells stably expressing CFP-tagged wild type (*bottom panel*) or dDAT-G108Q (*top panel*), incubated in the absence (*control lane*) and presence of noribogaine (30 μm), pifithrin-μ (5 μm), and the combination of noribogaine and pifithrin-μ for 24 h. After electrophoretic separation and transfer to nitrocellulose membranes, immunoreactivity of dDAT-G108Q was detected via its N-terminal tag with an antibody directed against GFP (*upper blot*). *C* and *M* indicate the position of the core-glycosylated (ER resident) and mature (Golgi and post-Golgi) forms of dDAT-G108Q, respectively. *c,* detergent lysates were prepared from HEK293 cells stably expressing CFP-tagged dDAT-G108Q, as described under “Experimental Procedures.” *Left panel,* transporter was immunoprecipitated with an anti-GFP antibody, and immunoreactive bands were detected with appropriate antibodies directed against GFP (for DAT), HSP70, and calnexin (*CNX*). *Right panel,* association between dDAT-G108Q and chaperones was quantified using ImageJ 1.43 from four independent experiments. The ratios were normalized to control; the *error bars* represent S.E. The statistical comparison was done by repeated measures analysis of variance followed by Bonferroni's multiple comparisons using the original rather than the normalized values (*p* < 0.05 significantly different from wild type, *p* value for HSP70 = 0.0053 and for calnexin = 0.0017).

We also examined whether the combination of noribogaine and pifithrin-μ exerted an additive action; HEK293 cells stably expressing dDAT-G108Q were treated with increasing concentrations of noribogaine (0–30 μm) in the presence or absence of pifithrin-μ (5 μm). Preincubation with noribogaine resulted in a concentration-dependent increase in [^3^H]dopamine uptake by the mutant (*closed squares* in Fig. 5*a*). In the presence of pifithrin-μ (*open circles* in Fig. 5*b*), the pharmacochaperoning effect of noribogaine was enhanced. Thus, the two compounds acted in an additive manner. We confirmed that the enhanced [^3^H]dopamine uptake seen after preincubation with noribogaine and/or pifithrin-μ arose from an increased ER export of dDAT-G108Q by examining the relative proportion of core-glycosylated ER resident and the mature glycosylated form; it is evident from Fig. 5*b* that cells, which had been pretreated noribogaine or pifithrin-μ, produced a larger amount of mature core glycosylated band of dDAT-G108Q mutant than untreated control cells. Consistent with the data shown in Fig. 5*a*, the combination of noribogaine and pifithrin-μ produced the largest increase in the mature core glycosylated form.

Pretreatment of cells with noribogaine and pifithrin-μ is predicted to reduce the association of dDAT-G108Q with calnexin and HSP70-1A, because stalled folding intermediates are chaperoned along the folding trajectory to a stable minimum energy conformation, which can be subsequently exported from the ER. We verified this prediction by immunoprecipitating dDAT-G108Q from cells that had been treated with noribogaine or pifithrin-μ. In fact, either manipulation increased the total amount of dDAT-G108Q, which was recovered by immunoprecipitation (*cf. top immunoblot* in Fig. 5*c*), but it reduced the relative amount of HSP70-1A and calnexin, which were found in complex with dDAT-G108Q (*lower two immunoblots* in Fig. 5*c*). The combination of noribogaine and pifithrin-μ again resulted in the largest effect on both the total amount of dDAT-G108Q and on complex formation with calnexin and HSP70-1A (*right-hand lanes* in Fig. 5*c* and *right-hand bars* in Fig. 5*c*).

##### Restoration of Axonal Targeting and of Sleep in Fruit Flies Harboring hDAT-G140Q and dDAT-G108Q

**FIGURE 6.**
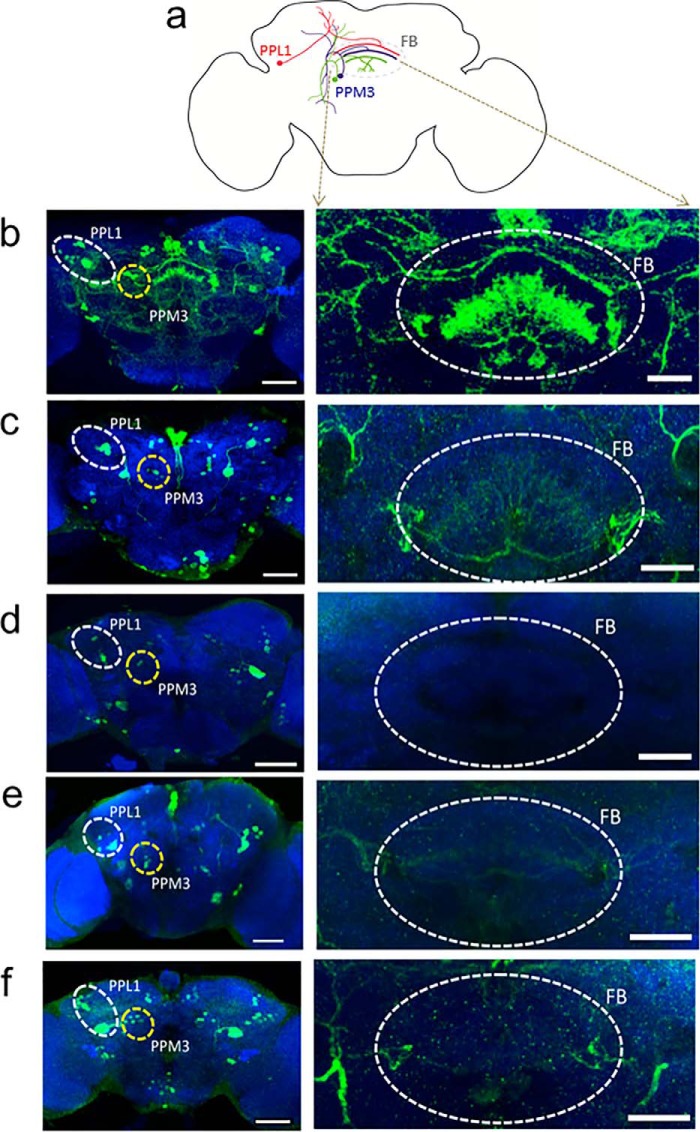
**Effect of pharmacochaperoning on trafficking of the hDAT-G140Q mutant.** *a,* schematic of dopaminergic neurons innervating the fan-shaped body (*FB*) in adult fly brain. Single dopaminergic neuron from PPL1 cluster (marked in *red*) innervate the dorsal fan-shaped body. Two dopaminergic neurons from PPM3 cluster (marked in *blue* and *green*) innervate medial and ventral fan-shaped body. *b–f, left-* and *right-hand panels* show confocal images of the posterior half and a section through the fan-shaped body of the mounted fly brains, respectively. Neurons were visualized by staining with the nc82 antibody (*blue background*); the expression of GFP- or YFP-tagged constructs in PPM3 (dorsomedial posterior protocerebral neurons) and PPL1 (dorsolateral posterior protocerebral neurons) was visualized with an antibody against GFP (*green* staining). Shown are representative brain sections of flies (TH-GAL4 driver lines) expressing GFP-mCD8 (*b*), YFP-tagged UAS-hDAT (*c*), YFP-tagged UAS-hDAT-G140Q (*d*), and of flies expressing YFP-tagged UAS-hDAT-G140Q (*e*), which had received noribogaine (30 μm) via their food from the larval stage or of adult flies expressing YFP-tagged UAS-hDAT-G140Q (*f*), which were fed noribogaine (30 μm) for 7 days and underwent behavioral monitoring (*cf*. Fig. 7). Each image is representative of at least 10 additional images per condition. *Scale bars* for the *left panels* represent 50 μm and for *right panels* 20 μm.

The average sleep time of *D. melanogaster* is 16 h (13). Although screening the “Zuker collection” of viable *Drosophila* mutants (21), Wu *et al.* (13) identified a mutant fly that slept for ∼5 h and had a point mutation of glycine 108 to glutamine in DAT. The sleep time of the flies carrying this point mutation (from here on referred to as dDAT-G108Q flies) is similar to that of flies, in which DAT had been knocked out (14). The experiments summarized in Figs. 4 and 5 showed that pharmacochaperoning partially restored ER export of and rescued dopamine transport by dDAT-G108Q in transfected HEK293 cells. Hence, we reasoned that pharmacochaperoning *in vivo* ought to restore sleep in these flies provided that the mutated transporter reached the presynaptic terminals. In *Drosophila*, dopaminergic innervation of the “fan-shaped body” neuropil in the central brain is critical for sleep regulation (22). The dopaminergic projections originate from the protocerebral posterolateral (PPL1) neurons and protocerebral posteriomedial cluster 3, PPM3 (22). We employed the GAL4/UAS system (23) to express YFP-tagged versions of wild-type hDAT (UAS-hDAT) and of hDAT-G140Q (UAS-hDAT-G140Q) in dopaminergic neurons using a tyrosine hydroxylase GAL4 (TH-GAL4) driver line (24). We also expressed a GFP-tagged version of murine CD8 (UAS-mCD8) under the control of TH-GAL4. CD8 is a membrane protein. When expressed under the control of TH-GAL4, mCD8 allows for identifying dopaminergic neurons; this is illustrated in Fig. 6*b* for the somata of PPL1 and PPM3 neurons (Fig. 6*b*, *left-hand image*) and their axonal projections (*FB* in the *right hand image* of Fig. 6*b*), which terminate in the dorsal, medial, and ventral portion of the fan-shaped body, respectively (22). YFP-tagged wild-type hDAT was also visualized in both the cell bodies of PPL1 and PPM3 neurons and their presynaptic terminals within the fan-shaped body (Fig. 6*c*). In contrast, YFP-tagged hDAT-G140Q was confined to the PPL1 and PPM3 cell bodies; here, the presynaptic terminals in the fan-shaped body were devoid of any detectable fluorescence (Fig. 6*d*). However, detectable amounts of hDAT-G140Q did enter the axonal compartments and reached the presynaptic sites in the fan-shaped body, if the flies were fed noribogaine starting from first instar larval stage until adulthood (Fig. 6*e*). Based on this observation, we also explored whether administration of the pharmacochaperone to adult flies sufficed to promote folding and subsequent ER export of hDAT-G140Q. This was the case; in adult flies, which had been exposed to noribogaine via the food for 5 days, YFP-tagged hDAT-G140Q was visualized in the fan-shaped bodies (Fig. 6*f*), albeit at substantially lower levels than YFP-tagged wild-hDAT (*cf*. Fig. 6, *f* and *c*).

**FIGURE 7.**
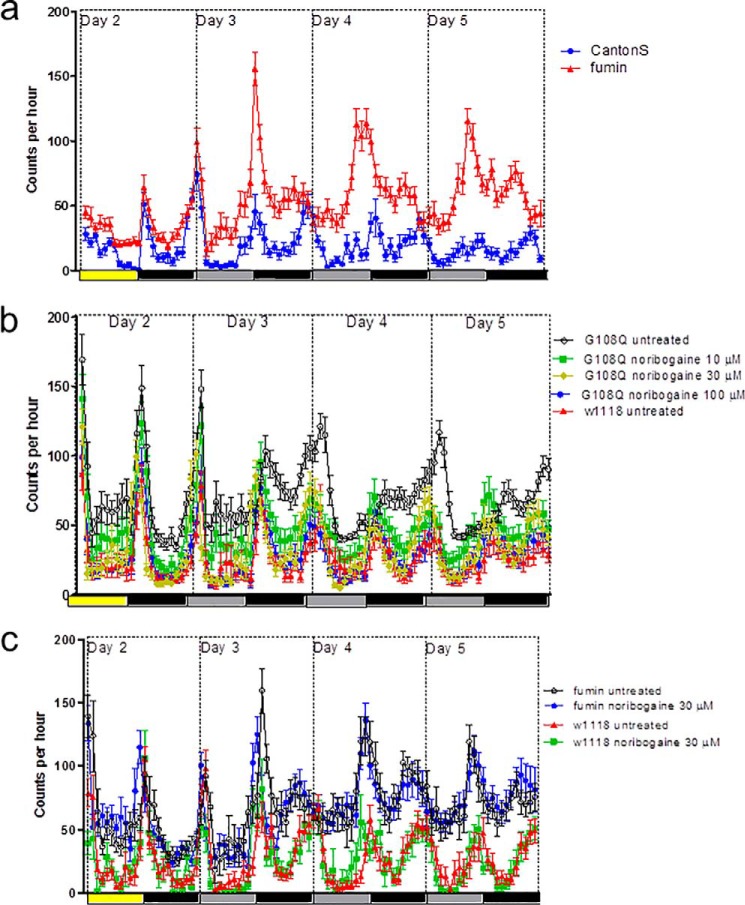
**Locomotor activity of dDAT-deficient *fumin* flies (*a* and *c*), Canton S control flies (*a*), dDAT-G108 flies (*b*), and w1118 control flies (*b* and *c*).** Locomotion of individual flies was recorded at 1-min time intervals using a *Drosophila* activity monitoring system (Trikinetics) for 7 days; beam crossings are summarized in 60-min bins (data from day 1 are not shown because flies were allowed to recover from CO_2_ anesthesia; data from days 6 and 7 have been omitted because the circadian rhythm became progressively desynchronized). After day 2 (12-h light/12-h dark cycle indicated by *yellow* and *black bars*), the flies were switched to 12-h dark/12-h dark cycles (indicated by the *gray* and *black bars*). *b* and *c*, flies received the indicated concentrations of noribogaine in the food pellet (*i.e.* 10, 30, and 100 μm, *green, red,* and *blue symbols/lines* in *b*, respectively; 30 μm in *c*). Data are means ± S.E. from three independent experiments, which were carried out in parallel with 10 flies per condition.

We verified that the amount of mutant DAT, which reached the axonal territory in the fan-shaped body, sufficed to restore sleep in dDAT-G108Q flies. In these flies the mutant DAT is under the control of the endogenous promoter. Several fly lines were used as reference, including the DAT-deficient *fumin* flies, which are hyperactive (14), and cn1bw1 (*i.e.* the original line mutagenized to obtain dDAT-G108Q flies), w1118, and Canton S, which have a normal sleep length. Three- to 5-day-old male flies were placed individually into a monitoring system, which allowed for recording their locomotor activity. On day 1 (12-h light/12-h dark cycle) flies were allowed to recover from the CO_2_ anesthesia. On day 2, the circadian rhythm was entrained by a 12-h light/12-h dark cycle. Subsequently, the flies were switched to a 12-h dark/12-h dark cycle) and observed for 5 additional days. It is evident from Fig. 7*A* that the difference in activity level of dDAT-deficient fumin flies and of Canton S control flies was magnified, when the animals were on a 12-h dark/12-h dark cycle. This is consistent with the finding that light exposure suppresses the wake-promoting action of dopamine (25). Accordingly, we focused on the 12-h dark/12-h dark cycles to examine the ability of noribogaine and of pifithrin-μ to restore sleep in dDAT-G108Q flies. The individually housed flies received food, which had been fortified with increasing concentrations of noribogaine and pifithrin-μ. The original activity traces are shown in Fig. 7*b* for untreated dDAT-G108Q flies (*black traces*), which were hyperactive, when compared with w1118 flies (*red traces*). The locomotor activity of dDAT-G108Q flies, which had received increasing amounts of noribogaine via their food, was reduced in a dose-dependent manner; in fact, dDAT-G108Q flies treated with 100 μm noribogaine in the food pellet (*blue traces* in Fig. 7*b*) were indistinguishable from w1118 (*black traces* in Fig. 7*b*). In contrast, neither the locomotor activity of fumin flies nor of w1118 flies was affected by 10–100 μm noribogaine in the food pellet (shown for 30 μm noribogaine in Fig. 7*c*). It is also evident from Fig. 7*b* that the effect of noribogaine on the activity of dDAT-G108Q flies was maintained over the entire observation period.

**FIGURE 8.**
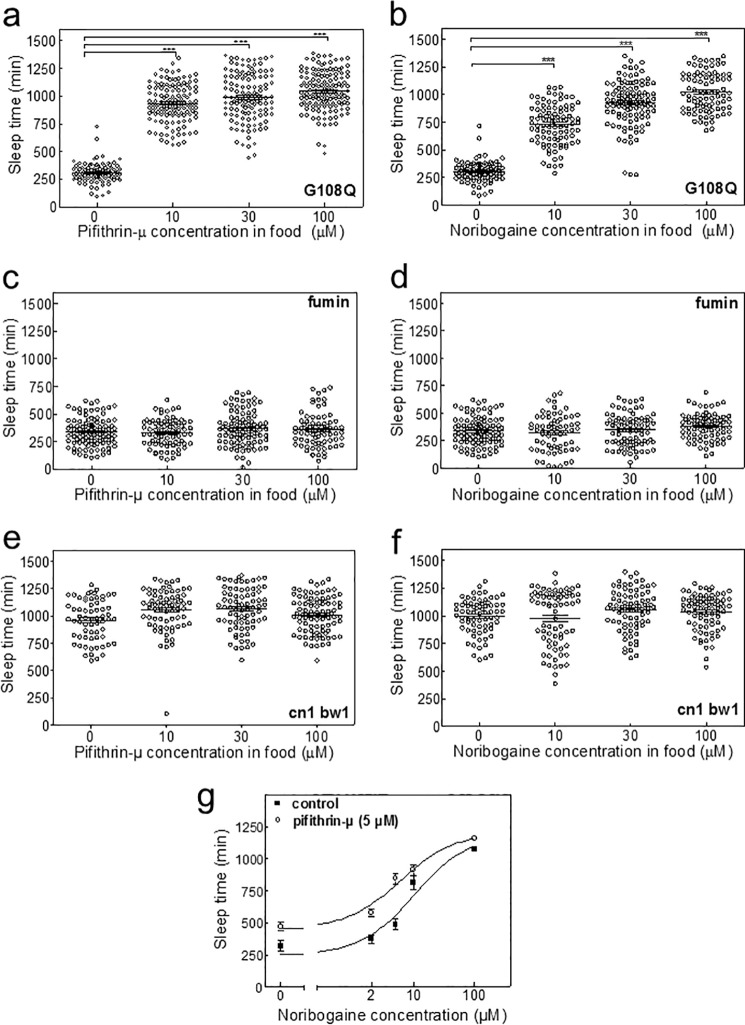
***In vivo* pharmacochaperoning restores sleep in flies carrying the dDAT-G108Q mutation.** Male flies with the homozygous genotypes dDAT-G108Q (*a, b,* and *g*), dDAT-KO (*c* and *d*), and cn1bw1(*e* and *f*) received pifithrin (*a, c,* and *e*), and noribogaine (*b, d* and *f*) or the combination of both (*g*) as outlined under “Experimental Procedures.” The total amount of sleep in the flies was calculated for each condition. Means ± S.E. are indicated. The experiments shown in *a* and *b* and *c* and *d* were done in parallel. Accordingly, the controls (0 drug in food pellet) are the same. They are shown to illustrate the effect of pifithrin and noribogaine (*a* and *b*) and the absence of any effect (*c* and *d*) in flies harboring DAT-G108Q and in fumin flies, respectively. *Symbols* in *a–f* correspond to recordings of individual flies, which were obtained in five independent experiments, where 8–25 flies were observed/condition. The statistical comparison was done by analysis of variance followed by Dunn's post hoc test (***, *p* < 0.001, significantly different from control).

Analogous experiments were carried also out with increasing amounts of pifithrin-μ (original traces not shown). Inactivity for 5 min or more was considered as sleep. As shown before (13), dDAT-G108Q flies slept only for around 300 min (Fig. 8, *a* and *b*), *i.e.* the time expected from dDAT-deficient *Drosophila* (14). If dDAT-G108Q flies ingested food containing pifithrin-μ (Fig. 8*a*) or noribogaine (Fig. 8*b*), an increase in total sleep time was observed. We used the locomotor activity recorded on day 4 to quantify fly sleep, but we stress that similar results were obtained, if the calculation was based on the entire period ranging from days 3 to 5. In contrast, neither pifithrin (Fig. 8*c*) nor noribogaine (Fig. 8*d*) affected the very short sleep time of dDAT-deficient fumin flies (Fig. 8, *c* and *d*). Hence, we rule out that these compounds had an effect on sleep time in the absence of any DAT, which could serve as substrate for pharmacochaperoning. Similarly, we examined cn1bw1 flies, which were originally used for random mutagenesis to create the Zuker collection. Sleep in these cn1bw1 flies is normal (on average about 1000 min). It was also not changed by the administration of noribogaine (Fig. 8*f*) and pifithrin-μ (Fig. 8*e*). Similar results were obtained in CantonS and w1118 flies, which were used as additional control (data not shown, *cf*. Fig. 7*c* for 30 μm noribogaine and w1118). These observations rule out that noribogaine or pifithrin-μ exerted their action by a nonspecific action, *i.e.* by effects unrelated to their ability to restore the function of dDAT-G108Q. Finally, we also verified that pharmacochaperoning restored sleep in flies harboring hDAT-G140Q (data not shown). The data summarized in Fig. 5 indicate that the combination of noribogaine and pifithrin-μ act in an additive manner to promote folding and the subsequent ER export of dDAT-G108Q. This was also recapitulated *in vivo*; if the concentration of pifithrin in the food was held constant at 5 μm, which as such elicited only a very modest increase in sleep time, the additional administration of noribogaine further promoted sleeping; pifithrin-μ shifted the dose-response curve of noribogaine to the left (*cf. open* and *closed symbols* in Fig. 8*g*; half-maximum enhancement of sleep was seen at 9.9 ± 1.3 and 6.0 ± 1.2 μm noribogaine in food for the flies, which received noribogaine only and the combination of noribogaine and pifithrin-μ, respectively).

## Discussion

Diseases that arise from misfolding of an individual protein are rare. However, collectively, these folding diseases represent a large proportion of hereditary and acquired disorders. In fact, the origin of molecular medicine can be traced to the study of a folding disease, *i.e.* sickle cell anemia (26). Folding diseases arising from mutations in SLC6 transporters have been identified only recently (5). Here, we capitalized on a serendipitous mutation, which had been introduced into the gene encoding DAT of *D. melanogaster,* to provide a proof-of-principle for pharmacochaperoning in a living organism. Our findings show that the sleepless phenotype of dDAT-G108Q harboring flies can be accounted for by a folding defect of the mutant protein. This conclusion is based on four independent lines of evidence as follows. (i) Molecular dynamics simulations showed that the G108Q mutation impaired the ability of TM12 to adopt a stable conformation. (ii) In transfected cells, dDAT-G108Q (and its human counterpart) was visualized in the endoplasmic reticulum rather than at the cell surface; accordingly, it failed to mediate cellular uptake of its substrate. Similarly, dDAT-G108Q accumulated as the ER retained core-glycosylated species, whereas the mature complex-glycosylated form was virtually absent. (iii) Large amounts of dDAT-G108Q were trapped in complex with calnexin and HSP70-1A. This association directly reflects stalling of the mutant along the folding trajectory, because calnexin and HSP70-1A act as endogenous folding sensors. (iv) The folding defect of dDAT-G108Q was overcome by incubating the cells with the pharmacochaperone noribogaine or by the HSP70 inhibitor pifithrin-μ (19); complex formation with calnexin and HSP70-1A was reduced, and dDAT-G108Q acquired the mature glycan structure and reached the cell surface such that dopamine transport became detectable in dDAT-G108Q expressing cells. Pharmacochaperoning was also effective *in vivo*; in noribogaine-treated flies dDAT-G108Q reached the axonal projections to a level that sufficed to restore sleep.

An important role in the structure of the transporter can be inferred from the fact that the glycine residue equivalent to Gly^108^ of dDAT is absolutely conserved in all SLC6 family members. This glycine is juxtaposed to the first intracellular loop IL1 and the third transmembrane domain (TM3). Earlier work indicated that IL1 was important for surface expression of the norepinephrine transporter (27–29). In fact, the segment of IL-1 adjacent to TM3 communicates with the C terminus; in SERT, folding requires the formation of a salt bridge between IL-1 and the end of the C-terminal α-helix (10). In dDAT, this communication is achieved by a cation-π interaction (15). Glycine 108 is part of a G*XXX*G-related motif, which stabilizes the interaction between TM3 and TM12 at the intracellular leaflet of the membrane. The size of the glutamine side chain at position 108 is too large to be accommodated by the G*XXX*G motif. Thus, helix TM12 was displaced in all simulations, which started from the folded dDAT and carried the G108Q mutations. This observation indicates that the mutation interferes with packing of helix TM12 in the folding trajectory of dDAT. This has repercussions for the position of the C-terminal helix, because it closely follows TM12. During folding of SERT in the ER, HSP70 engages the proximal segment of the SERT C terminus. Mutations in the C terminus result in SERT variants, which are stalled in this HSP70-bound state, and thus fail to progress in the folding trajectory; the energy barrier can be lowered and folding of these SERT mutants induced by addition of noribogaine or by introducing second site suppressor mutations, which trap SERT in the inward-facing conformation (8). It is plausible to infer a similar mechanism for DAT. Because of this communication between IL1 and the C terminus, it is possible to rationalize why the pharmacochaperone noribogaine and the HSP70 inhibitor pifithrin-μ (19) acted in an additive fashion to restore folding, surface expression, correct targeting, and functional activity of dDAT-G108Q. SLC6 transporters must engage their cognate SEC24 isoform to reach the axonal compartment (30–32). Recruitment of SEC24 is contingent on prior release of heat-shock proteins from the C terminus of the folded transporter (5, 8, 10). It is evident from the molecular dynamics simulations that in 1 out of 6 simulations the water entry into the hydrophobic core and the hydration of Gln^108^ did not occur in dDAT-G108Q (*cf*. Fig. 1*d*). This suggested that dDAT-G108Q could, albeit rarely, also reach a point that precludes the entry of destabilizing water. Pharmacochaperoning may work by allowing the folding trajectory to progress through this or a related conformational species.

Our findings have implications that go beyond remedying sleep deficiency in mutant flies. First, at least 10 mutations occur in human DAT, which give rise to a syndrome of infantile or juvenile dystonia and parkinsonism (2, 3, 33). Thus, the present proof-of-principle delineates an approach that may be suitable for rescuing folding defective DAT in affected individuals. Second, it is worth noting that a mutation of the corresponding glycine has been observed in the human creatine transporter-1 (hCrT1, SLC6A8); the mutation (resulting in hCrT1-G132V) is associated with severe mental retardation and autistic behavior (34). Our observations indicate that the phenotype also results from a folding deficit and hence the loss-of-function phenotype of the creatine transporter-1.^5^ Hence, we consider it likely that this deficiency can also be remedied by pharmacochaperoning. However, despite the rich pharmacology, *i.e.* the very large number of available ligands available (1), the only ligands that we have found to act as pharmacochaperones are ibogaine and noribogaine. This is presumably related to the fact that these compounds trap the monoamine transporters in the inward-facing state (11, 12). The current evidence indicates that the folding trajectory of SLC6 transporters progresses through the inward-facing conformation (8–10). The vast majority of SLC6 inhibitors bind to the outward facing state; compounds that bind preferentially to the inward-facing state of SLC6 family members are apparently rare, with the only examples being ibogaine and noribogaine. Thus, the finding that the inhibitor of HSP70 pifithrin-μ (19) rescues the mutants dDAT-G108Q and hDAT-G140Q may also be of relevance for developing an alternative therapeutic strategy to restore surface expression of the misfolded CrT1.

## Experimental Procedures

### 

#### 

##### Homology Modeling and Simulations

Homology models of dDAT were built using MODELLER version 9.15 (17). The thermostabilizing mutants V74A and L415A were mutated back to the wild-type sequence. The EL2 loop was truncated for crystallization, but this modification did not affect transporter function (16). We maintained the truncated EL2 sequence because no template for the full loop of dDAT was available. We replaced the conformation of the EL2 loop of the crystal structure with the loop conformation developed for the human DAT (18). This substitution was necessary because the EL2 loop extracted from the crystal structure was unstable when its interactions with the co-crystallized antibody in the adjacent crystallographic unit were removed. We created 100 models of wild-type and mutant dDAT and selected the best three models based on the DOPE score (35). The selected models were inserted into a pre-equilibrated POPC membrane and electroneutralized by adding the appropriate number of ions of a 150 mm NaCl solution. We also exchanged residue 108 between glycine and glutamine in all six systems after membrane insertion to control for the possibility that subtle details of the dDAT model or the membrane insertion affected the results. Every system was independently equilibrated, and MD simulations were carried for 100 ns. Simulations were carried out with the Gromacs simulations package, version 4.6.6 (36). The POPC membrane was described using the Berger lipid parameters (37), and the AMBER force field (38) was used for the dDAT. Temperature was maintained at 310 K using the v-rescale (τ = 0.1 ps) thermostat (39) by independently coupling protein, membrane, and solvent to a reference temperature bath. Pressure was maintained at 1 bar using the Berendsen barostat (40) using a pressure coupling constant of 1.0 ps, and the compressibility was 4.5 × 10^−5^ bar^−1^. The van der Waals interactions were described using the Lennard Jones potential applying a cutoff of 1.0 nm. Long range electrostatic interactions were calculated using the smooth particle mesh Ewald method (41) with a cutoff of 1.0 nm. The water molecules were constrained using the SETTLE algorithm (42), although all other bonds were constrained by LINCS (43). Long range corrections for energy and pressure were applied.

##### Chemicals

[^3^H]Dihydroxyphenylethylamine(dopamine; Long range corrections for energy 36.6 Ci/mmol) and [^3^H]CFT (76 Ci/mmol) were purchased from PerkinElmer Life Sciences. Cell culture media, supplements, and antibiotics were from Invitrogen. Bovine serum albumin (BSA) and Complete^TM^ protease inhibitor mixture were from Roche Applied Science (Mannheim, Germany); SDS was from BioMol GmbH (Hamburg, Germany); Tris and scintillation mixture (Rotiszint® eco plus) were from Carl Roth GmbH (Karlsruhe, Germany). Anti-GFP antibody (ab290) was from Abcam Plc (Cambridge, UK). Protein A-Sepharose and anti-rabbit IgG1-antibody linked to horseradish peroxidase were from Amersham Biosciences. All other chemicals were of analytical grade. The 0.4% trypan blue stock solution was purchased from Sigma. Pifithrin was purchased from Sigma. Noribogaine was purchased from Cfm Oskar Tropitzsch GmbH (Marktredwitz, Germany).

##### Drosophila Genetics

The dDAT G108Q mutant flies (Bloomington stock no. 30867) and cn1 bw1flies (Bloomington stock no. 264) were ordered from Bloomington *Drosophila* stock center, Bloomington, IN. Fumin flies (DAT-KO mutant flies) were the generous gift of Dr. Kume, Nagoya City University, Japan. YFP-tagged hDAT and hDAT-G140Q were cloned into pUAST-attB vector (gift from Drs. Bischof and Basler, University of Zürich). The constructs were injected into embryos from M{vas-int.Dm}ZH-2A, M{3×P3-RFP.attP0}ZH-86Fb (Bloomington stock no 24749). Positive transformants were isolated and selected. All flies were kept at 25 °C, and all crosses were performed at 25 °C. The genotypes of flies were as follows: +/+; +/+; *TH-Gal4/UAS-mCD8GFP* (Fig. 6*b*), +/+; +/+; *TH-Gal4/UAS-hDAT* (Fig. 6*c*), +/+; +/+; *TH-Gal4/UAS-hDAT-G140Q* (Fig. 6, *d–f*), cn^1^ DAT^Z2–1744^ bw^1^ (Fig. 7*b*; Fig. 8, *a*, *b* and *g*), w; fmn (w; roo{}DAT^fmn^) (Fig. 7, *a* and *c*; Fig. 8, *c* and *d*), cn^1^ bw^1^ (Fig. 8, *e* and *f*). dDAT-G108Q and fumin flies were isogenized by crossing with balancer flies (Bloomington stock no. 3704).

##### Mutagenesis, Cell Culture, and Transfection

Mutations were introduced into plasmids encoding YFP-hDAT or CFP-dDAT using the QuikChange Lightning site-directed mutagenesis kit (Agilent Technologies, Santa Clara, CA). The mutagenic primers were designed using the QuikChange primer design tool provided by the manufacturer. HEK93 cells were grown at 37 °C in a 5% CO_2_ humidified atmosphere, in Dulbecco's modified Eagle's medium (DMEM), which had been supplemented with 10% fetal calf serum and penicillin (60 mg/liter) and streptomycin (100 mg/liter). The cells were transfected using Lipofectamine 2000 (Life Technologies, Inc.), according to the protocols provided with the reagents. After 48 h, the cells were transfected with plasmids (0.5–2 μg/10^6^ cells) encoding the transporters using Lipofectamine 2000 (Invitrogen). S2R+ cells (DGRC, Bloomington, IN) were grown at 25 °C, in Schneider's *Drosophila* medium (Invitrogen) that had been supplemented with heat-inactivated 10% fetal calf serum, penicillin (60 mg/liter), and streptomycin (100 mg/liter). S2R+ cells were transfected using Effectene transfection reagent (Qiagen, Limburg, Netherlands) according to the manufacturer's instructions (DNA/enhancer/Effectene ratio was 1 μg, 8 μl, 25 μl).

##### Radiotracer Assays and Confocal Microscopy

For uptake assays, transfected cells were seeded onto poly-d-lysine-coated 48-well plates to a density of ∼10^5^ cells per well. After 24 h, the cells were washed with Krebs-HEPES (KRH) buffer (10 mm HEPES, 120 mm NaCl, 3 mm KCl, 2 mm CaCl_2_, 2 mm MgCl_2_, 2 mm glucose monohydrate, 1 mm tropolone, 10 μm pargyline, and 1 mm ascorbic acid, pH 7.3) and incubated for 5 min with 0.2 μm [^3^H]dopamine, at room temperature. Uptake was terminated by rapid wash with ice-cold KRH buffer. For experiments where *K_m_* and *V*_max_ values were determined, [^3^H]dopamine was diluted with unlabeled dopamine to achieve final concentrations of 0.2, 1, 3, 10, 15, and 30 μm. Background uptake was determined in the presence of 30 μm mazindol (44). Uptake in S2R+ cells was performed in tubes, because the cells grow partially in suspension. Uptake was terminated by rapid washing with ice-cold buffer, and the cells were collected by filtration.

##### Whole Cell Binding Assays

HEK293 cells transiently expressing wild-type or mutant transporters were incubated with [^3^H]CFT in a total reaction volume of 0.2 ml of KRH buffer containing 10 μm ZnCl_2_, in the presence or absence 30 μm mazindol, to determine nonspecific binding. The reactions were incubated for 15 min and were terminated by rapid washing with ice-cold buffer and collected onto glass fiber filters (Whatman GF/B) that were dissolved in scintillation medium and counted for radioactivity.

For imaging, HEK293 cells expressing wild-type CFP-hDAT or CFP-dDAT or DAT mutants and S2R+ cells expressing CFP-dDAT and dDAT mutants were seeded onto poly-d-lysine-coated ibidi® glass bottom chambers. After 24 h, cell imaging was performed. Confocal microscopy was done using a Zeiss LSM780 equipped with an argon laser (at 30 milliwatts) and a ×63 oil immersion objective (Zeiss Plan-Neofluar). The plasma membrane was delineated by staining with trypan blue, and ER was outlined by plasmid-driven expression of YFP-calnexin (8, 10). In the case of S2R+ cells, ER was marked by cherry-KDEL, and myristoylated and palmitoylated YFP was co-transfected with CFP-tagged versions of dDAT to label the cell membrane.

##### Co-immunoprecipitation and Cell Surface Biotinylation

Immunoprecipitation and detection of complexed proteins was done as described earlier (8–10). Briefly, adherent DAT-expressing cells were washed with PBS twice, mechanically detached in lysis buffer (50 mm Tris-HCl, pH 8.0, 150 mm NaCl, 1% dodecyl maltoside, 1 mm EDTA, and the Complete^TM^ protease inhibitor mixture), and collected by centrifugation. Detergent-insoluble material was removed by centrifugation (16,000 × *g* for 30 min at 4 °C). Equal amounts of protein (2 mg/sample) were incubated for 16 h with 4 μl of anti-GFP antibody (20 μg IgG). Subsequently, pre-equilibrated protein A-Sepharose (20 mg of protein A/sample) was added and incubated at 4 °C for 5 h with gentle rotation. The beads were collected by centrifugation and then washed three times with lysis buffer. Bound proteins were eluted by denaturation in 0.1 ml of sample buffer containing 40 mm dithiothreitol and 1% mercaptoethanol at 45 °C for 30 min. An aliquot from the original cellular lysate was also denatured in sample buffer, and aliquots were resolved by PAGE. Proteins were transferred onto nitrocellulose membranes, which were blocked with 5% bovine serum albumin in Tris-buffered saline containing 0.1% Tween 20 (TBST). Membranes were incubated with antibodies directed against GFP, calnexin, or HSP70-1A in TBST overnight at 4 °C. Subsequently, membranes were washed in TBST, and horseradish peroxidase-(HRP)-conjugated secondary antibody (1:5000) was applied, and the immunoreactivity was detected by chemiluminescence. Biotinylation of cell surface proteins was done as described previously (45). Briefly, HEK cells were incubated in the presence of sulfo-NHS-SS-biotin (sulfosuccinimidyl-2-(biotinamido)ethyl-1,3-dithiopropionate; Pierce; 1 mg/ml). After cell lysis, the labeled surface proteins were retrieved by binding to streptavidin beads (Thermo Fisher Scientific Inc.). The amount of biotinylated DAT was quantified by immunoblotting. The G protein β-subunit was visualized by immunoblotting with an antibody directed against the N terminus (46) to verify that comparable amounts of lysate had been employed. Immunoreactive bands were quantified based on sensitive chemiluminescent detection (FluorChem HD2 system, Alpha Innotech; San Jose, CA) in the auto-acquired imaging mode.

##### Immunohistochemistry and Imaging

Adult fly brains were dissected in phosphate-buffered saline (PBS) and fixed in 2% paraformaldehyde in PBS for 1 h at room temperature. After fixation, brains were washed three times in 0.1% Triton X-100 in PBS for 20 min. Blocking was performed in 10% goat serum for 1 h at room temperature. Brains were then incubated in primary antibody overnight in PBS containing 3% BSA and 0.3% Triton X-100 at 4 °C. The mouse monoclonal antibody nc82 (anti-Bruchpilot; 1:30 dilution; DSHB, University of Iowa) and rabbit polyclonal IgG directed against GFP (1:1000 dilution; A-11122, Invitrogen) were used as primary antibodies. After three 20-min washes with PBS containing 0.1% Triton X-100, the brains were incubated overnight at 4 °C with secondary antibody in PBS containing 0.3% Triton X-100. Alexa Fluor 488 goat anti-rabbit IgG (1: 500; Invitrogen) and Alexa Fluor 647 goat anti-mouse IgG (1:500; Invitrogen) were used as secondary antibodies. Following incubation with secondary antibody, the brains were washed three times with PBS containing 0.1% Triton X-100 and were mounted using Vectashield® (Vector Laboratory, Burlingame, CA). Images were captured on a Leica SP5II confocal microscope with 20-fold magnification. Z-stack images were scanned at 1-μm section intervals with a resolution of 1024 × 1024 pixels. Images were processed with ImageJ and Adobe Photoshop CS5.1.

##### Behavioral Assays and Treatments of Flies

Flies were entrained to a 12:12 h light/dark cycle. Three- to five-day-old male flies were used for activity monitoring using the DAM2 *Drosophila* activity monitor system (Trikinetics, Waltham, MA). Eight to 25 flies were used per experimental condition, and the experiments were reproduced in five independent experiments. Flies were individually housed in 5-mm diameter glass tubes, which contained the food pellet with the indicated concentrations of noribogaine and/or pifithrin-μ, which were freshly dissolved in water and mixed with regular food (5% sucrose in 1% agarose). Flies were kept on a 12:12 h light/dark cycle for the first 2 days. From day 3 on, the cycle was shifted to a 12:12 h dark/dark for 5 additional days. Flies received the indicated drugs throughout the study period, which lasted for 7 days. The locomotion activity was constantly monitored for 7 days. Data from the 2nd day of the dark/dark phase was used for calculations. Activity was measured in 1-min bins, and the pySolo software (47) was used to quantify fly sleep. Inactivity of a fly for 5 min or more was considered as sleep. Flies received normal food (5% sucrose in 1% agarose) prior to their individual housing. All behavioral experiments were performed at 25 °C.

## Author Contributions

M. F., A. K., T. H., and S. S. designed the experiments and wrote the paper. A. E. K. and S. S. performed and analyzed the experiments shown in Figs. 2, 3, and 5. A. E.-K. and H. M. M. A. performed and analyzed the experiments shown in Fig. 3. A. K. performed and analyzed the experiments shown in Figs. 6–8. A. G. provided technical assistance. D. S. and T. S. did the MD simulations. All authors reviewed the results and approved the final version of the manuscript.

## Footnotes

5 A. El-Kasaby, S. Sucic, and M. Freissmuth, unpublished observations.

## Footnotes

4 The abbreviations used are:

DAT: dopamine transporter
DA: dopamine
CFP: cyan fluorescent protein
CT-1: creatine transporter 1
dDAT: *Drosophila* dopamine transporter
hDAT: human dopamine transporter
ER: endoplasmic reticulum
IL-1: intracellular loop 1
SLC6: solute carrier 6
SERT: serotonin transporter
TBS: Tris-buffered saline
r.m.s.d.: root mean square displacement
TM: transmembrane
MD: molecular dynamics
mut: mutant
POPC: 1-palmitoyl-2-oleoyl-l-phosphatidylcholine.
